# Research on a Silicon Gyroscope Interface Circuit Based on Closed-Loop Controlled Drive Loop

**DOI:** 10.3390/s22030834

**Published:** 2022-01-22

**Authors:** Qiang Li, Lifeng Ding, Xiaowei Liu, Qiang Zhang

**Affiliations:** 1 Shanxi Key Laboratory of Micro Nano Sensors & Artificial Intelligence Perception, College of Information and Computer, Taiyuan University of Technology, Taiyuan 030024, China; liqiang02@tyut.edu.cn; 2 Key Lab of Advanced Transducers and Intelligent Control System of the Ministry of Education, Taiyuan University of Technology, Taiyuan 030024, China; 3 Department of Chemistry and Chemical Engineering, Taiyuan Institute of Technology, Taiyuan 030008, China; dinglf@tit.edu.cn; 4 MEMS Center, Harbin Institute of Technology, Harbin 150001, China; lxw@hit.edu.cn

**Keywords:** gyroscope, closed-loop controlled drive loop, linear model, complementary metal oxide semiconductor (CMOS)

## Abstract

The existing analysis methods for the silicon gyroscope drive loop, such as the perturbation method and period average method, cannot analyze the dynamic characteristics of the system. In this work, a linearized amplitude control model of the silicon gyroscope drive loop was established to analyze the stability and set-up time of the drive loop, and the vibration conditions of the silicon gyro were obtained. According to the above results, a new silicon gyroscope interface circuit was designed, using a 0.35 μm Bipolar-CMOS-DMOS (BCD) process, and the chip area was 4.5 mm × 4.0 mm. The application-specific integrated circuit (ASIC) of the silicon gyroscope was tested in combination with the sensitive structure with a zero stability of 1.14°/hr (Allen). The test results for the ASIC and the whole machine prove the correctness of the theoretical model, which reflects the effectiveness of the stability optimization of the closed-loop controlled drive loop of the silicon gyroscope circuit.

## 1. Introduction

Gyroscopes are sensors that can be used to detect angular velocities [1,2,3]. They are widely used in science, technology, the military, and other fields [4,5,6]. Compared with traditional gyroscopes, silicon gyroscopes based on microelectromechanical system (MEMS) technology and CMOS technology have the characteristics of low cost, small size, low power consumption, high reliability, and mass producibility [7,8]. The silicon gyroscope interface circuit is required, to bring the gyroscope structure into resonance, and the angular velocity can be detected using the Coriolis force principle. Hence, closed-loop control drive loops based on automatic gain control (AGC) were commonly used to maintain the constant amplitude vibration of sensitive structures at their resonant frequencies [9,10]. In the closed-loop drive circuit of a silicon gyroscope, due to the highly non-linear component in the acceleration-to-speed signal transfer function, it was difficult to obtain an accurate analytical solution for the loop-transfer function [11,12].

In some traditional methods, such as the perturbation method and the period average method, the system was linearized near its equilibrium point and its time domain was analyzed, so the starting conditions of the system could be easily obtained. Nevertheless, it was difficult to obtain the specific performance of the system using traditional methods because their nonlinear functions were solved in the time domain and their solutions were too complex to obtain their analytical solutions [13,14]. Some works proposed models to simplify the amplitude response of a second-order system as a first-order system to analyze the stability of the system [15], but they did not quantitatively analyze the system response influenced by different control parameters.

In this work, the second-order transfer function of the drive loop was simplified in the low-frequency range to the first-order transfer function by using the perturbation term equivalent method, and an equivalent model of the silicon gyro drive loop was established. Based on this model, the characteristics of the amplitude frequency, phase frequency, and step response were simulated by SIMULINK, and parameters such as *K_i_* and *K_p_* were optimized according to the simulation results. According to the optimized parameters, the pre-stage circuit was adjusted. In order to verify the correctness of the model, a test system was established. The system’s start-up time and set-up time with different proportional integral controller (PI) parameters were compared by a transient response experiment for the silicon gyroscope. The performance of the silicon gyroscope interface circuit chip was tested and analyzed. According to the Allen variance method, the bias stability was 1.14°/hr, which met the requirements for high-precision silicon gyroscope sensors.

## 2. Drive Loop Modeling and Simulation

**Figure 1:**
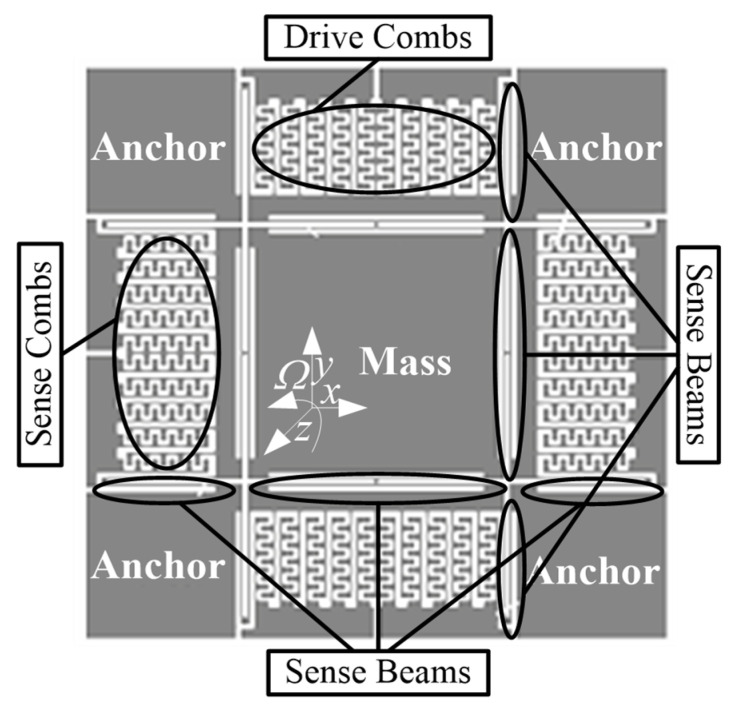
Sensitive structure of electrostatically driven capacitive silicon gyroscopes.

The electrostatically driven capacitive silicon gyroscope was analyzed as an example. Its operating principle is shown in Figure 1.

### 2.1. Mechanical Motion Principle of Silicon Gyroscope

Silicon gyroscopes measure angular velocity based on the Coriolis Force effect. When there is an angular velocity input perpendicular to the direction of a silicon gyroscope resonance plane, a forced vibration is generated in its resonance plane perpendicular to the resonance direction, and the input angular velocity can be calibrated by measuring its forced vibration. Equation (1) is the expression for the Coriolis Force [16]:

(1) $$\vec{F}=2m(\vec{\Omega }\times \vec{v})$$

where *m* is the effective mass of the structure motion, $\vec{\Omega }$ is the input instantaneous angular velocity, and $\vec{v}$ is the velocity of the structure motion.

Let the direction of the vibration of the silicon gyroscope driving the modal mass block be the X-axis and the direction of the vibration of the detecting modal mass block be the Y-axis. When the whole gyroscope rotates in the Z-axis with angular velocity *Ω*, the Coriolis Force is generated in the Y-axis direction. When the silicon gyroscope mass block is subjected to simple harmonic forces $F_{0}\sin\omega_{d}t$ in the driving direction, the dynamics equations for the gyroscope in the driving and detecting modes can be expressed as Equations (2) and (3) [17]:

(2) $$M_{d}\frac{d^{2}x}{dt^{2}}+\lambda_{d}\frac{dx}{dt}+K_{d}\cdot x=F_{0}\sin\omega_{d}t$$

(3) $$M_{s}\frac{d^{2}y}{dt^{2}}+\lambda_{s}\frac{dy}{dt}+K_{s}y=2M_{s}\Omega x^{\prime }(t)$$

where $M_{d}$ is the mass of the driving mass, and $M_{s}$ is the mass of the sensing mass. $\lambda_{d}$ and $\lambda_{s}$ are the damping force coefficients of the mass in the X-axis and Y-axis directions. $K_{d}$ and $K_{s}$ are the elasticity coefficients of the mass in the directions of the X-axis and Y-axis. *x* and *y* are the displacement of the mass in the X- and Y-axis directions.

### 2.2. The Establishment of the Closed-Loop Control Drive-Loop Model

In the drive loop of the silicon gyroscope, the transfer function of the detection structure that converts the acceleration into the velocity signal has a highly nonlinear component, and it is difficult to obtain an accurate analytical solution [18,19].

The kinetic equation of the drive mode in the driving velocity control gyroscope can be described as a deformation of Equation (2):

(4) $$\ddot{x}+2\xi_{d}\omega_{d}\dot{x}+\omega_{d}{}^{2}x={\dot{u}}_{x}$$

where $\omega_{d}=\sqrt{K_{d}/M_{d}}$ is the intrinsic frequency of the drive mode, $\xi_{d}=\lambda_{d}/2M_{d}\omega_{d}$ is the damping ratio of the drive mode, and the drive signal of the gyroscope is represented by the driving acceleration $u_{x}$, which could be obtained as *k_v_ux* in the driving loop, where *k_v_* is a displace to voltage conversion gain, *u* is the controller voltage.

The analytical solution could be written approximately as:

(5) $$x(t)\approx a(t)\sin(\omega_{x}t+\theta (t))$$

where *a*(*t*) and *θ*(*t*) are the time-varying amplitude and phase. $\omega_{x}=\omega_{d}\sqrt{1-\xi_{d}{}^{2}}$. The first and second derivatives of *x*(*t*) could be written as:

(6) $$\dot{x}=\dot{a}\sin(\omega_{x}t+\theta )+a\cos(\omega_{x}t+\theta )(\omega_{x}+\dot{\theta })$$

(7) $$\ddot{x}=\ddot{a}\sin(\omega_{x}t+\theta )+2\dot{a}\cos(\omega_{x}t+\theta )(\omega_{x}+\dot{\theta })-a\sin(\omega_{x}t+\theta ){\left(\omega_{x}+\dot{\theta }\right)}^{2}+a\cos(\omega_{x}t+\theta )\ddot{\theta }$$

Substituting Equations (5)–(7) into Equation (4) [20], one can derive:

(8) $$\begin{array}{l} \ddot{a}\sin(\omega_{x}t+\theta )+2\dot{a}\cos(\omega_{x}t+\theta )(\omega_{x}+\dot{\theta })+a[\cos(\omega_{x}t+\theta )\ddot{\theta }-\sin(\omega_{x}t+\theta ){\left(\omega_{x}+\dot{\theta }\right)}^{2}]\\ +2\xi_{d}\omega_{d}[\dot{a}\sin(\omega_{x}t+\theta )+a\cos(\omega_{x}t+\theta )(\omega_{x}+\dot{\theta })]+\omega_{d}{}^{2}a\sin(\omega_{x}t+\theta )\\ =k_{v}u[\dot{a}\sin(\omega_{x}t+\theta )+a\cos(\omega_{x}t+\theta )(\omega_{x}+\dot{\theta })] \end{array}$$

Considering the cosine term, one can derive:

(9) $$\dot{a}+\xi_{d}\omega_{d}a+\frac{\ddot{\theta }}{2(\omega_{x}+\dot{\theta })}a=\frac{1}{2}k_{v}ua$$

Since the resonant frequency of the sensor was several kilohertz, compared to *x_d_ω_d_*, the disturbance term $\ddot{\theta }/2(\omega_{x}+\dot{\theta })$ could be ignored. *k_v_ua* is the envelope signal of *u_x_*, which could be redefined as *u_a_*. Therefore, the transfer function could be rewritten as:

(10) $$G^{\prime }(s)=\frac{a(s)}{u_{a}(s)}=\frac{1}{2(s+\xi_{d}\omega_{d})}$$

This could mean that the second-order transfer function of the original drive loop could be simplified in the low-frequency range to the first-order transfer function that only described the output signal.

**Figure 2:**
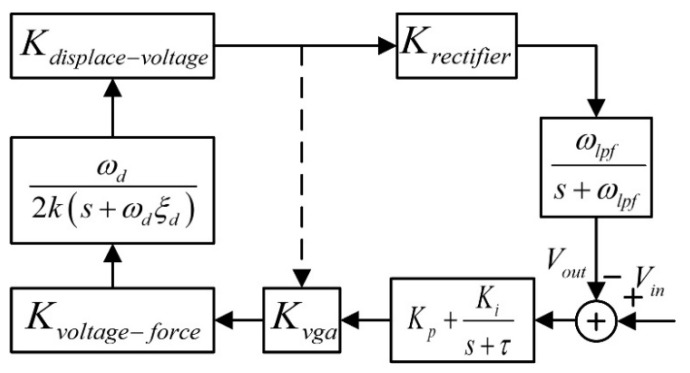
Closed-loop model of gyroscope drive loop.

Based on the analysis above, the closed-loop model of the gyroscope drive loop shown in Figure 2 could be established. As shown in the figure, in order to find the transfer function of the drive loop, the reference voltage input *V_ref_* was used as the input, and the output of the low-pass filter was used as the output. The closed-loop transfer function of the entire loop system could be obtained as:

(11) $$\frac{V_{out}}{V_{in}}=\frac{K_{total}K_{vga}V_{ref}V_{dc}\frac{\omega_{d}}{2k\left(s+\omega_{d}\xi_{d}\right)}\frac{\omega_{lpf}}{s+\omega_{lpf}}\left(K_{p}+\frac{K_{i}}{s+\tau }\right)}{1+K_{total}K_{vga}V_{ref}V_{dc}\frac{\omega_{d}}{2k\left(s+\omega_{d}\xi_{d}\right)}\frac{\omega_{lpf}}{s+\omega_{lpf}}\left(K_{p}+\frac{K_{i}}{s+\tau }\right)}$$

where *K_vga_* is the gain of the variable gain amplifier, *V_dc_* is the driving direct current (DC) voltage, *k* is the spring constant, *ω_lpf_* is the cutoff frequency of the filter, *K_p_* and *K_i_* are the proportional and integral terms of the PI controller, and *K_total_* is the product of *K_voltage-force_*, *K_displace-voltage_*, and *K_rectifier_*.

### 2.3. Simulation Result of the Model

According to Figure 2 and Equation (8), a SIMULINK simulation model was established, and the influence of *K_i_*, *K_p_*, *ω_lpf_*, and *K_vga_* on the system’s amplitude–frequency characteristics and unit step response was analyzed.

**Figure 3:**
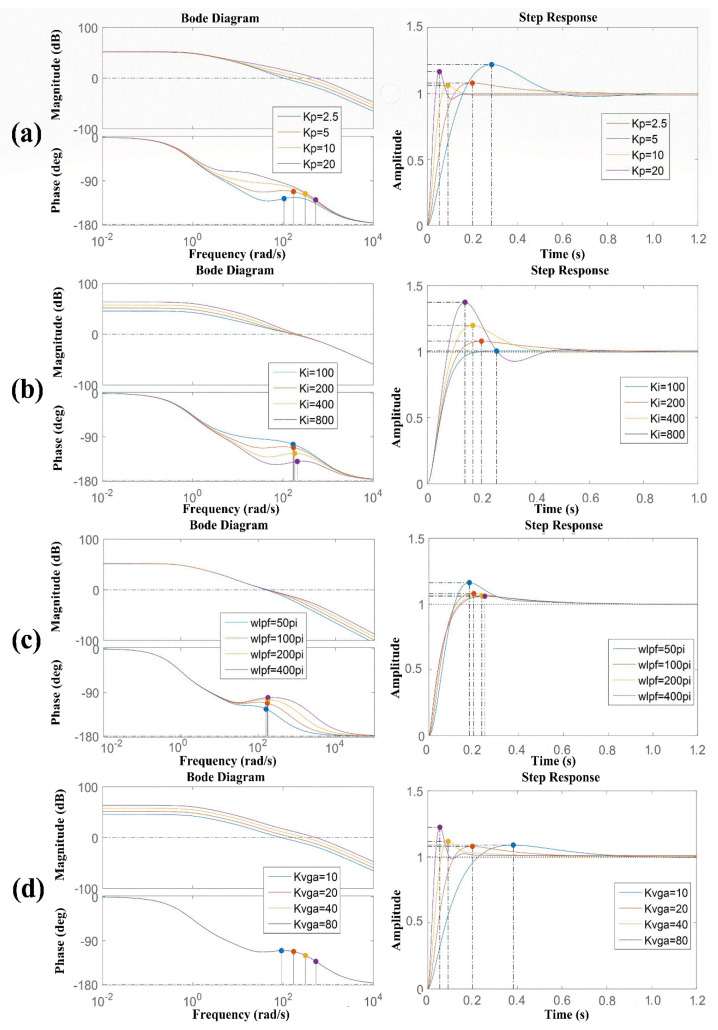
Amplitude frequency characteristics of the driving loop when (**a**) *K_p_*, (**b**) *K_i_*, (**c**) *ω_lpf_*, and (**d**) *K_vga_* changed.

As shown in Figure 3a, with an increase in *K_p_*, the gain of the system remained unchanged, and the bandwidth increased. The setup time was the shortest when *K_p_* = 10. Therefore, considering the system comprehensively, *K_p_* = 10 was the optimal value for the system parameters. As shown in Figure 3b, the loop gain increased when *K_i_* increased, but the bandwidth did not change much. According to the step response of the system, *K_i_* = 200 was a suitable value. In Figure 3c, it can be observed that the cut-off frequency of the low-pass filter could be chosen. It could be observed that the cut-off frequency of the low-pass filter had little effect on the gain and bandwidth of the system, and the step response indicated that the choice of *ω_lpf_* should not be too small. As shown in Figure 3d, an increase in *K_vga_* would significantly increase the system gain and bandwidth.

**Figure 4:**
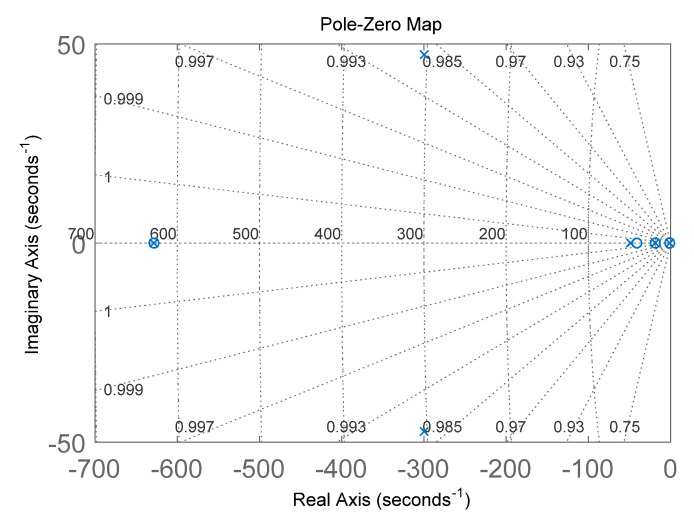
Schematic diagram of the zero pole of the drive loop.

In addition, the zero pole of the system could also be observed in the root trajectory diagram of the closed-loop system, as shown in Figure 4.

Therefore, a strategy for optimizing the system parameters for this structural parameter could be derived from the results of Figure 3 and Figure 4. In order to obtain drive-loop parameters with better stability and robustness, a shorter build-up time, and less system oscillation, appropriately increasing *K_i_* and *K_vga_* to obtain a larger system gain, and then adjusting the value of *K_p_* to change the zero point of the complex plane and the loop stability, could be considered.

## 3. Circuit Design and Experiments

**Figure 5:**
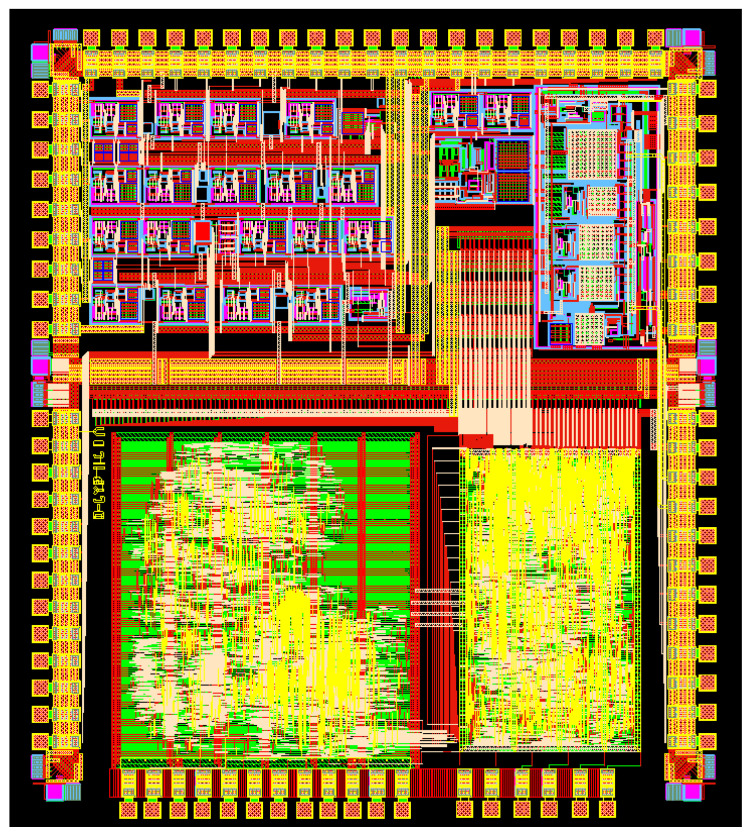
Chip layout of silicon gyroscope interface circuit.

The 0.35 μm four-metal double polycrystalline N-well CMOS process was used to complete the layout design of the silicon gyroscope interface ASIC chip. Figure 5 shows the layout of the interface circuit chip.

### 3.1. Overall Design of the Drive Loop

**Figure 6:**
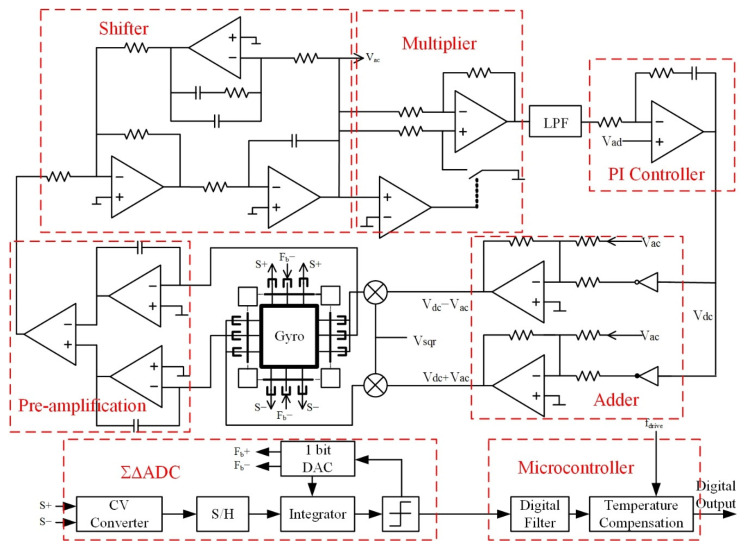
Overall design of silicon gyroscope interface circuit.

In the drive loop of Figure 6, the signal of the drive detection in the gyroscope structure was detected using a charge amplifier. After differencing, amplification, phase shifting, and demodulation, the signal with the same resonant frequency of the drive mode was obtained, and the automatic gain control of this signal was realized through a peak detection module and PI control module. The final drive signal was superimposed with the DC reference signal, which was applied to the drive combs at the top and bottom of the left and right sides of the gyroscope structure to complete the self-excited drive of the silicon gyroscope.

### 3.2. Circuit Implementation Details

#### 3.2.1. Charge–Voltage (CV) Conversion Circuit

**Figure 7:**
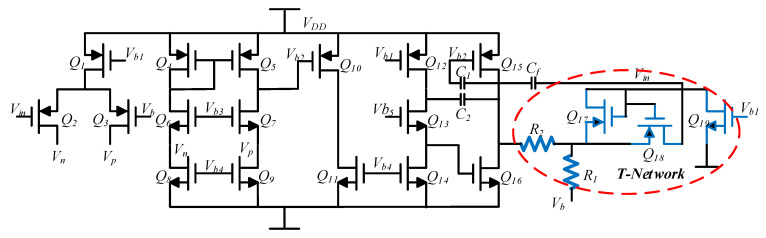
Charge amplifier circuit.

Figure 7 shows the structure of the three-stage operational amplifier circuit used in the root preamplifier circuit. A T-network structure was used in this operational amplifier to implement a large resistor to increase the transimpedance gain and reduce noise. In addition, using this T-network structure could increase the integration and reduce the chip area. This T-shaped transimpedance network consisted of a transistor and a resistor in the red circle, which could achieve an equivalent feedback resistance greater than 100 MΩ. The principle of this T-shaped resistor network was to make the gate source voltages of the two transistors equal. *Q_17_* and *Q_19_* were set so that *Q_18_* was in the linear region and had a larger equivalent resistance due to its smaller gate source voltage and smaller aspect ratio. This equivalent resistance was proportional to the bias resistance in the bias current source and was not affected by time and temperature variations [21]. The feedback capacitor *C_f_* was about 5 pF, and its resistance was matched to the silicon gyroscope sensitive structure to reduce the effect of parasitic capacitance. The equivalent resistance of the T-shaped network was:

(12) $$R_{eq}=R_{M}(1+\frac{R_{2}}{R_{1}})+R_{2}$$

where *R_M_* was the equivalent resistance of the transistor *Q*_18_, and its resistance was about 1 MegΩ, which was much larger than *R*_1_ and *R*_2_.

#### 3.2.2. Phase-Compensation Circuit

**Figure 8:**
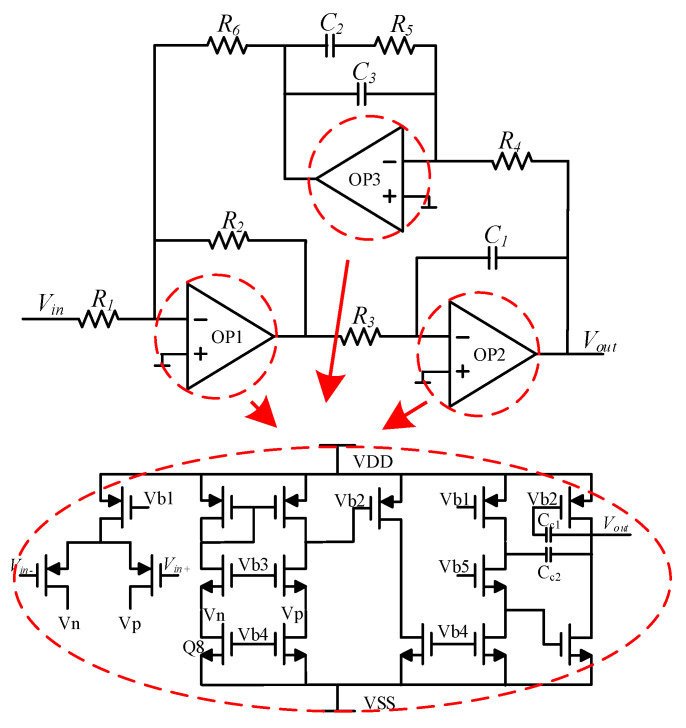
Diagram of phase shifter.

Figure 8 shows a block diagram of the phase-compensation circuit, i.e., phase shifter. Phase shift was generated in the pre-stage CV conversion, so the phase had to be shifted by 90° in the post-stage circuit to meet the phase conditions of the closed-loop self-excited drive. The operational amplifier *OP*_1_ and the resistors *R*_1_, *R*_2_, and *R*_6_ formed an adder, wherein the resistance values of *R*_1_, *R*_5_, and *R*_6_ were equal, to realize negative feedback. The operational amplifier *OP*_2_, the resistor *R*_3_, and the capacitor *C*_1_ formed a feedforward integrator. The operational amplifier *OP*_3_, resistor *R*_5_, and capacitors *C*_2_ and *C*_3_ formed a feedback integrator. A forward transfer integrator was used to achieve a 90° phase shift, and a feedback integrator was used to eliminate the continuously integrated forward transfer integrator detuning voltage.

#### 3.2.3. Automatic Gain Control Circuit

**Figure 9:**
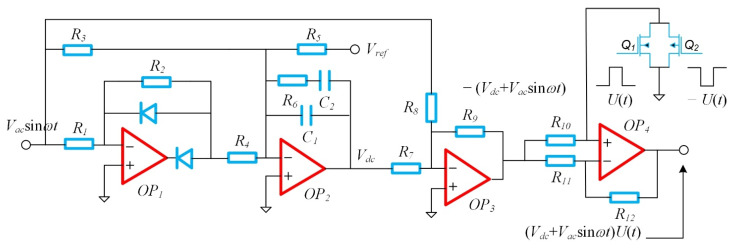
Principle diagram of the automatic gain-control and modulation drive circuit.

Figure 9 shows a schematic diagram of the automatic gain control and drive modulation circuit, which was used to adjust the DC bias of the closed-loop drive voltage to achieve a dynamic amplitude stabilization drive.

As shown in Figure 9, the half-wave rectifier circuit consisted of an operational amplifier *OP*_1_, and resistors *R*_1_ and *R*_2_ with two diodes; the full-wave rectification and low-pass filtering functions were completed with an integrator composed of the operational amplifier *OP*_2_ and *C*_1_, *C*_2_ and *R*_6_, and the resistors *R*_3_ and *R*_4_. The inverting input of the integrator was connected to the voltage reference source through the resistor *R_5_*, and the integrator completed the closed-loop amplitude control function. The parameters in the circuit were *R*_1_ = *R*_2_, *R*_3_ = 2*R*_4_, *R*_7_ = *R*_8_ = *R*_9_, and *R*_10_ = *R*_11_ = *R*_12_. After the closed-loop feedback, the integrator automatically adjusted the output DC voltage *V_dc_*; the alternating current voltage amplitude, *V_ac_*, was:

(13) $$V_{ac}=\frac{\pi R_{3}V_{ref}}{2R_{5}}$$

The adder consisted of the operational amplifier *OP*_3_ with the resistors *R*_7_, *R*_8_, and *R*_9_, whose function was to superimpose the signals *V_dc_* and *V_ac_*. The multiplier consisted of the operational amplifier *OP*_4_ with the transistors *Q*_1_ and *Q*_2_, whose function was to complete the high frequency modulation of the driving voltage signal, with a function of (*V_dc_* + *V_ac_*sin*wt*)*U*(*t*), to avoid coupling interference. In the multiplier, a switch consisted of the transistors *Q*_1_ and *Q*_2_, whose gates were controlled by voltage square waves ±*U*(*t*) with a period of *T_S_* = 25 ms and a duty cycle of 50%, which was used to realize the square wave modulated signal.

### 3.3. Verification of Closed-Loop Control Drive-Loop Model

**Figure 10:**
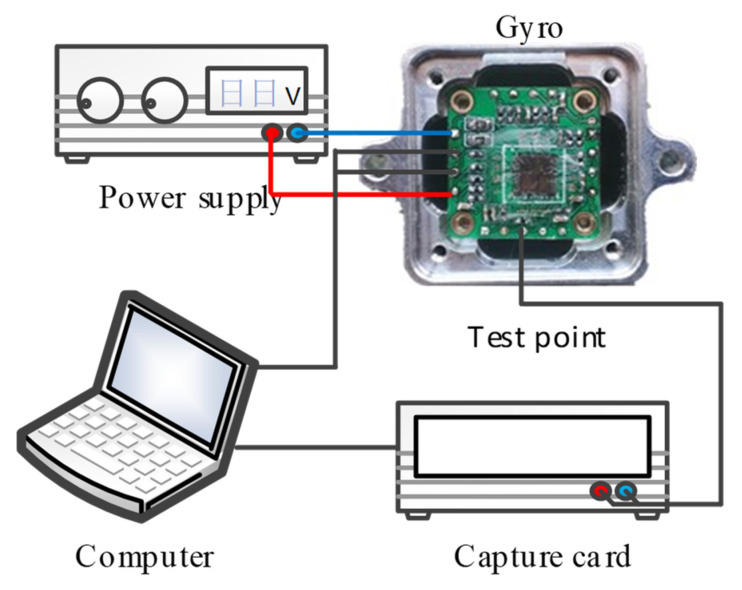
Stability test system for closed-loop controlled drive loop.

**Figure 11:**
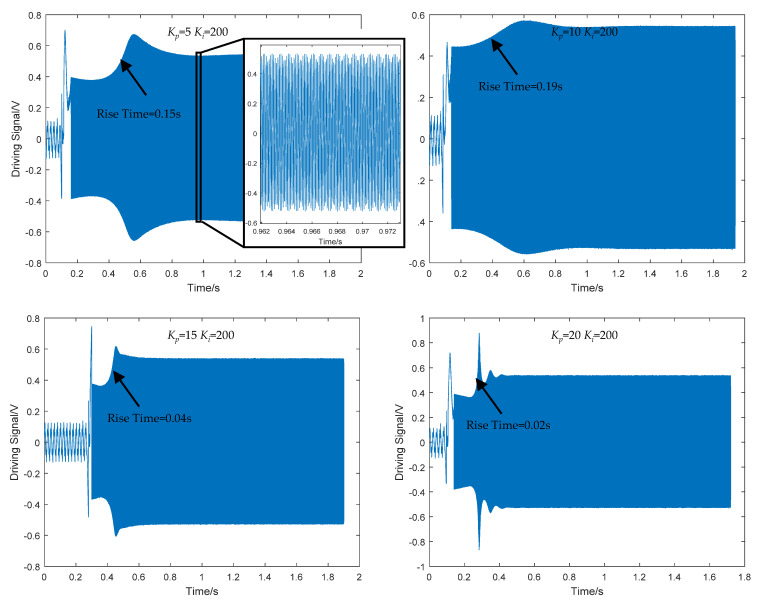
Transient response of PI parameters in closed-loop controlled drive loop.

In order to verify the correctness of the stability analysis and stability model of the drive loop in this work, a transient response experiment for the silicon gyroscope was carried out, and the system’s start-up time and set-up time were mainly compared when using different PI parameters. In order to test the transient response of the gyroscope drive loop, a test system was established, as shown in Figure 10. Keysight’s U2355A high-speed data acquisition card was used to capture the signal at a sampling frequency of 50 kHz, and the debug interface was used to switch the power of the entire interface circuit on and off to generate a step signal. After the system was powered on, the drive signal was sampled, and the sampled result was processed by Matlab. The transient response of the closed-loop drive circuit when the PI controller started to oscillate with different parameters is shown in Figure 11.

When the drive signal was started using different *K_p_*, the transient waveforms were as shown in Figure 11. It could be observed that within about 0.2 s when the silicon gyroscope system was powered on, the driving loop did not start immediately, and the driving signal had not yet been established. Then the noise components were continuously selected and amplified by the closed-loop self-excited driving loop through frequency selection, a tiny driving signal was generated which was rapidly amplified by the multiplier. After a period of rise time, it was quickly stabilized at a fixed amplitude under the action of the closed-loop oscillation automatic gain control module in the driving loop as a sine wave. It could be observed that with *K_p_* increasing from 5 to 10, the overshoot signal was gradually smoothed, and the rise time and settling time were increased. When *K_p_* continued to be increased, the overshoot signal was increased again. The test result proved that, when *K_p_* = 10 and *K_i_* = 200, the stability optimization of the control loop was realized.

**Figure 12:**
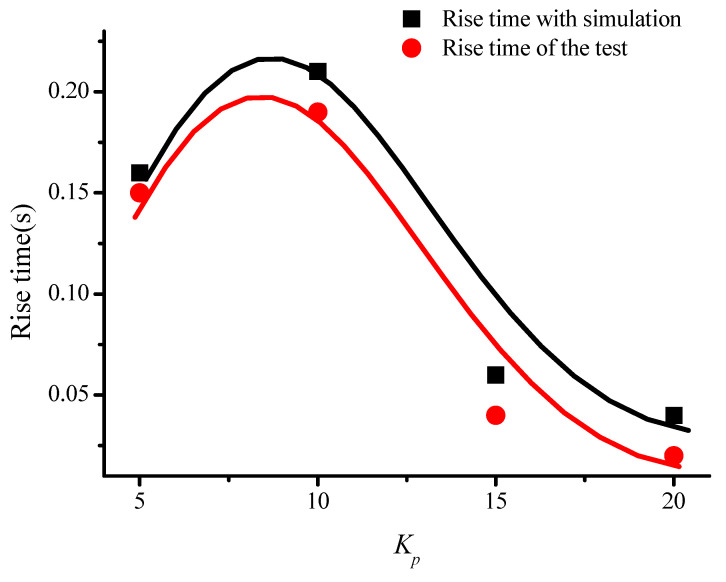
Comparison of simulation and test results when *K_p_* changed.

In order to prove the conclusion above, the comparison between the simulation and the test about rise time was given in Table 1 and Figure 12. It could be observed that the rise time of the model was almost the same as the test result. Compared with the test results, the rise time of the drive-loop model was slightly different. However, the change trends were the same, which also verified the correctness of the model.

### 3.4. Experimental Results for the Whole System

The system was tested by connecting the PAD points on the interface ASIC chip to the solder joints on the corresponding PCB with silicon aluminum wire through a press welder and integrating the ASIC chip on a PCB board. The circuit operated at a ±2.5 V supply voltage with a power consumption of 90mW. The main instruments and equipment used for the interface circuit testing are shown in Table 2.

**Figure 13:**
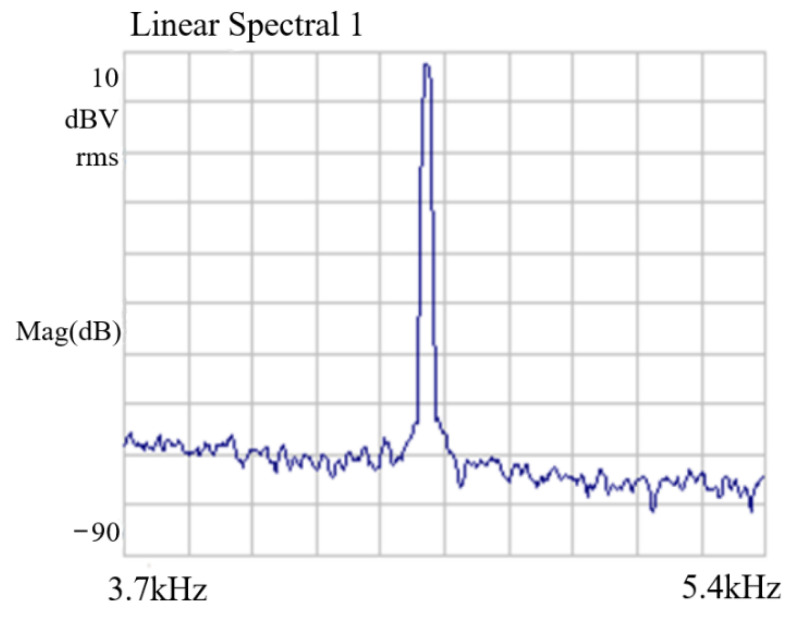
Spectrum of drive signal.

**Figure 14:**
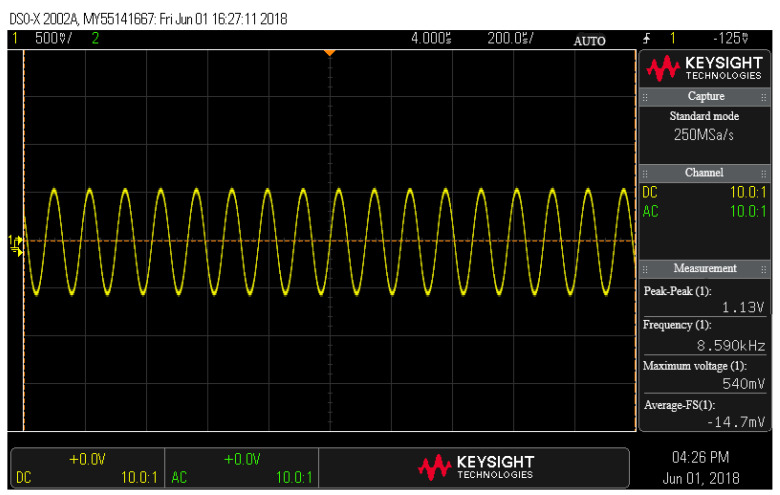
Time domain test chart for driving signal.

To verify the design of the self-excited drive circuit, the driving spectrum was analyzed using a dynamic analyzer, HP35670A. Figure 13 shows the spectrum of the drive signal of the closed-loop self-excited drive circuit. The unmodulated drive signal in the time domain was tested using an oscilloscope (DSOX2002A), and the test results are shown in Figure 14. The frequency stability was 0.93 ppm. This drive voltage signal was applied to the silicon gyroscope drive comb after high-frequency modulation. The test results show that the driving circuit could make the silicon gyroscope structure self-excited induce stable driving at the resonant frequency.

**Figure 15:**
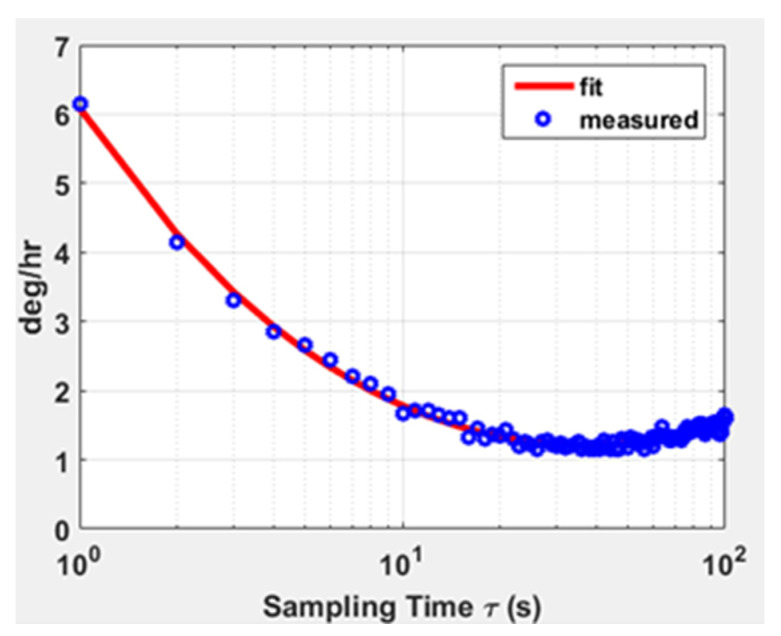
Zero output fitting curve for gyroscope generated using Allen variance.

A whole machine test on the silicon gyroscope was carried out. At room temperature, the serial debugging assistant was used to sample the digital output for one hour, and the sampled data were averaged every 10 s. The data results were fitted according to the international standard stability Allen variance method, as shown in Figure 15. The output bias stability was 1.14°/hr (Allen), which could meet the requirements for high-precision silicon gyro sensors. The effectiveness of the stability optimization of the drive loop was proved by the test results.

## 4. Discussion and Conclusions

In this work, a linearized amplitude control model of the silicon gyroscope drive loop was established. It solved the problem of the existing stability analysis methods for silicon gyroscope drive loops being unable to analyze the dynamic characteristics of the system. The model could be used to obtain the stability conditions of the system as well as to analyze the dynamic characteristics of the system. The correctness of the model was verified by comparing the experimental results with the simulation results. The proposed stability model could provide a theoretical basis for the optimization of the driving loop system parameters of the ASIC.

## Figures and Tables

**Table 1:** Test and simulation results of sensor with different proportional term *K_p_*.

*Kp*	5	10	15	20
Rise time with simulation (s)	0.16	0.21	0.06	0.04
Rise time of the test (s)	0.15	0.19	0.04	0.02

**Table 2:** Device used by silicon gyroscope sensor test system.

Equipment	Type	Manufacturer
High-precision current source	PW36-1.5ADP	KENWOOD
Current source	E3631A	Agilent
Dynamic signal analyzer	35670A	HP
Oscilloscope	DSOX2002A	Agilent

## Data Availability

Not applicable.
